# The Pituitary Gland of the European Eel Reveals Massive Expression of Genes Involved in the Melanocortin System

**DOI:** 10.1371/journal.pone.0077396

**Published:** 2013-10-10

**Authors:** Eirill Ager-Wick, Ron P. Dirks, Erik Burgerhout, Rasoul Nourizadeh-Lillabadi, Daniëlle L. de Wijze, Herman P. Spaink, Guido E. E. J. M. van den Thillart, Katsumi Tsukamoto, Sylvie Dufour, Finn-Arne Weltzien, Christiaan V. Henkel

**Affiliations:** 1 Department of Basic Sciences and Aquatic Medicine, Norwegian School of Veterinary Science, Oslo, Norway; 2 ZF-screens B.V., Leiden, The Netherlands; 3 Institute of Biology, Leiden University, Leiden, The Netherlands; 4 Atmosphere and Ocean Research Institute, the University of Tokyo, Kashiwa, Chiba, Tokyo, Japan; 5 Research Unit BOREA, Biology of Aquatic Organisms and Ecosystems, CNRS 7208, Muséum National d´Histoire Naturelle, Paris, France; Centre of Marine Sciences & University of Algarve, Portugal

## Abstract

Hormones secreted from the pituitary gland regulate important processes such as development, growth and metabolism, reproduction, water balance, and body pigmentation. Synthesis and secretion of pituitary hormones are regulated by different factors from the hypothalamus, but also through feedback mechanisms from peripheral organs, and from the pituitary itself. In the European eel extensive attention has been directed towards understanding the different components of the brain-pituitary-gonad axis, but little is known about the regulation of upstream processes in the pituitary gland. In order to gain a broader mechanistic understanding of the eel pituitary gland, we have performed RNA-seq transcriptome profiling of the pituitary of prepubertal female silver eels. RNA-seq reads generated on the Illumina platform were mapped to the recently assembled European eel genome. The most abundant transcript in the eel pituitary codes for pro-opiomelanocortin, the precursor for hormones of the melanocortin system. Several genes putatively involved in downstream processing of pro-opiomelanocortin were manually annotated, and were found to be highly expressed, both by RNA-seq and by qPCR. The melanocortin system, which affects skin color, energy homeostasis and in other teleosts interacts with the reproductive system, has so far received limited attention in eels. However, since up to one third of the silver eel pituitary’s mRNA pool encodes pro-opiomelanocortin, our results indicate that control of the melanocortin system is a major function of the eel pituitary.

## Introduction

The European eel (*Anguilla anguilla*) has a long and complex life cycle. It is now listed as a critically endangered species [1], leading to an urgent need to learn more about its biology and reproduction. Spawning of the European eel occurs in unknown areas of the Sargasso Sea [2]. Larvae drift to the European coasts following oceanic currents, where they metamorphose into glass eels and migrate into continental habitats. They can stay in brackish water or in rivers for years as juvenile yellow eels before they develop into prepubertal silver eels [3]. Silver eels are still sexually immature when they leave the continental habitats and migrate back to the sea. They remain blocked at the prepubertal stage as long as the reproductive migration is prevented [4]. Therefore, maturation needs to occur during the oceanic migration or at their spawning grounds [5]. The prepubertal silver eels are the last known stage of the eel life cycle in natural conditions. Although spawning European eels have never been caught in the wild, the spawning site of the Japanese eel (*Anguilla japonica*) was recently discovered close to the West Mariana Ridge [6]. Adults and newly hatched larvae were found in close proximity to the first collection of Japanese eel eggs.

Attempts to promote spontaneous maturation, gametogenesis and spawning of eels in aquaculture have so far been unsuccessful. In order to accomplish successful artificial reproduction, there is a need for development of strategies to control the secretion of hormones from the pituitary gland. To achieve this goal the endocrinological changes in the pituitary should be studied during maturation both at the transcriptome and proteome level. The pituitary comprises the neurohypophysis (posterior pituitary) and the adenohypophysis (anterior pituitary), both regulated by the hypothalamus. The adenohypophysis of teleosts is a major endocrine organ and is organized into different compartments, i.e. *rostral* and *proximal pars distalis* and *pars intermedia*, where different hormones are produced in their respective cell types [7–9]. The two gonadotropins, follicle-stimulating hormone and luteinizing hormone, directly control gonadal development. Other pituitary hormones, such as growth hormone and thyroid-stimulating hormone, regulate other physiological systems, but also play a role in reproduction. Adrenocorticotropic hormone, α- and β-melanocyte stimulating hormone and β-endorphin derive from a common precursor hormone, pro-opiomelanocortin. These hormones are important components of the melanocortin system, which is involved in the regulation of different physiological processes, and, in teleosts, possibly also in reproduction [10].

In order to get closer to understanding important molecular mechanisms at work in the European eel pituitary we have utilized high throughput RNA sequencing (RNA-seq) to profile the global gene expression in the pituitary of prepubertal silver eels. RNA-seq provides the opportunity to study the transcriptome in a specific organism, tissue or cell type by sequencing millions of short fragments simultaneously. The number of reads produced is a function of the abundance of a transcript, and thus the read density is used to quantify gene expression [11–13]. In addition to providing information about gene expression levels, RNA-seq also enables the discovery of new genes and transcripts and can reveal alternative splice isoforms [11,12,14].

The draft genomes of the European and the Japanese eel have recently been published [15,16], enabling for the first time reliable gene expression profiling using RNA-seq. However, curated gene annotations and expression data are still scarce for eels, and therefore the included gene predictions for the eel genome are primarily based on generic gene models [15–17]. In this study, we have therefore used RNA-seq evidence to manually improve the annotations of the most abundantly expressed genes in the eel pituitary.

## Materials and Methods

### Animals and experimental design

Female European eels (*Anguilla anguilla*) obtained in the Netherlands were used in this study. Four prepubertal eels (one wild and three farmed) in the silvering transition period were sampled for RNA-seq. To get closer to a complete picture of the European eel transcriptome we included one sample from two other developmental stages: a farmed immature yellow eel and a wild artificially matured eel. For qPCR validation of key findings we used pituitary material from five farmed silver eels, of which three were the same samples used for RNA-seq. Permission for capturing eels during the migration season was obtained from the Netherlands' Ministry of Agriculture and Fisheries. Experiments were approved by the animal ethical commission of Leiden University (DEC #08112 and #11093). The farmed animals (one yellow and three silver eels, samples 2–4) were transported from the farms to the University (1.5–2 h) in buckets with some water, and the pituitaries were sampled immediately upon arrival. The wild silver eel (sample 1) was kept for one week at the University in a seawater recirculation system prior to sampling. The artificially matured eel received weekly injections of salmon pituitary extract (20 mg) for 17 weeks and ovulation was induced by injection with 17, 20β-dihydroxy-4-pregnen-3-one (DHP) (2 mg kg^-1^) as recently published [18]. The matured eel was sampled just after ovulation. As an estimate of the reproductive status the gonadosomatic index (GSI) was calculated. Calculation of silver index, eye index and other morphological and physiological measurements were performed as previously described [19,20], and the detailed information about the animals is given in table 1. Prior to dissection of the pituitary gland, eels were euthanized using an overdose of anesthetic (clove oil), followed by decapitation. Eel pituitaries were sampled and stored in RNAlater (Ambion) at -80 °C until RNA extraction.

**Table 1:** Sample information.

**Sample**	**Silver eel 1**	**Silver eel 2**	**Silver eel 3**	**Silver eel 4**	**Silver eel 5**	**Silver eel 6**	**Yellow eel**	**Mature eel**
**RNA-seq**	yes	yes	yes	yes	no	no	yes	yes
**qPCR**	no	yes	yes	yes	yes	yes	no	no
**Sampling date**	07.12.2008	18.05.2011	18.05.2011	18.05.2011	18.05.2011	18.05.2011	27.08.2010	26.05.2010
**Sampling location**	Lake Grevelingen	Passie voor Vis BV	Passie voor Vis BV	Passie voor Vis BV	Passie voor Vis BV	Passie voor Vis BV	Nijvis Holding BV	Lake Veerse
**Country**	The Netherlands	The Netherlands	The Netherlands	The Netherlands	The Netherlands	The Netherlands	The Netherlands	The Netherlands
**Origin**	Wild (seawater)	Farmed (freshwater)	Farmed (freshwater)	Farmed (freshwater)	Farmed (freshwater)	Farmed (freshwater)	Farmed (freshwater)	Wild (seawater)
**Sex**	Female	Female	Female	Female	Female	Female	Female	Female
**BW**	1317.3	719.2	872.7	686.6	874.3	880.8	272.0	573.7
**BL**	88.4	67.2	69.9	70.2	72.6	71.6	50.2	77.9
**CF**	0.22	0.24	0.26	0.20	0.23	0.24	0.20	0.12
**EdH**	10.8	9.0	10.1	8.8	8.0	9.9	6.4	11.8
**EdV**	11.0	8.7	9.7	8.7	8.1	9.6	6.1	10.5
**EI**	10.6	9.2	11.0	8.6	7.0	10.4	6.1	12.5
**PFL**	42.4	24.3	24.4	27.7	27.8	28.4	17.5	35.6
**SI**	4	3	4	3	3	4	2	5
**LW**	13.2	4.1	8.0	4.2	8.1	8.0	4.2	9.9
**HSI**	1.00	0.57	0.92	0.61	0.93	0.91	1.54	1.73
**GW**	28.4	6.1	10.6	4.7	8.5	10.2	1.8	95.0
**GSI**	2.1	0.8	1.2	0.7	1.0	1.2	0.7	16.6

BW = body weight (g), BL = body length (cm), CF = condition factor, EdH = eye diameter horizontal (mm), EdV = eye diameter vertical (mm), EI = eye index (according to [19]), PFL = pectoral fin length (mm), SI = silver index (according to [20]), LW = liver weight (g), HSI= hepatosomatic index, GW = gonad weight (g), GSI = gonadosomatic index

### RNA extraction, Illumina library preparation and sequencing

Total RNA was isolated using the Qiagen miRNeasy kit according to the manufacturer’s instructions (Qiagen). RNA integrity was assessed by Agilent Bioanalyzer 2100 on a total RNA Nano series II chip (Agilent). All RNA-seq libraries were prepared with the Illumina mRNA-seq Sample Preparation Kit from 10 µg total RNA, according to the manufacturer’s instructions (Illumina Inc.). RNA-seq paired end libraries for silver eel 1, yellow eel, and mature eel were sequenced with a read length of 2×55 nucleotides on an Illumina GAIIx instrument, while the other silver eel samples (silver eel 2, 3 and 4) were sequenced with a read length of 2×51 nucleotides on a HiSeq2000 according to the manufacturer’s protocol. The Illumina pipeline was utilized for image analysis and base calling.

### Quantitative PCR

Quantitative PCR (qPCR) was carried out on a LightCycler 480 Real-Time PCR system (Roche, Mannheim, Germany), using the LightCycler 480 Master with SYBR Green (Roche). cDNA was prepared from 1 µg of DNase treated total RNA using Superscript III reverse transcriptase (Invitrogen) and oligo(dT) primers according to product specifications. To avoid amplification of contaminating genomic DNA, the primers were designed to span exon-exon boundaries, such that part of the primer hybridizes to the 3′ end of one exon and the rest of the primer hybridizes to the 5′ end of the adjacent exon. A standard dilution curve was set up for each primer pair and the pair that showed the best efficiency was chosen. These primer sequences are given in table S1. Acidic ribosomal phosphoprotein P0 (*arp*) shows stable expression during different experimental treatments in eel and was used as a reference gene to normalize the expression analysis [21], using an efficiency-corrected relative quantification method [22]. Each sample was analyzed in duplicate and comprised 5 µl mastermix, 2 µl primer mix (5 µM of each or forward and reverse), and 3 µl of each 10× diluted cDNA sample in a total volume of 10 µl. The cycling parameters were 10 min preincubation at 95 °C, followed by 42 cycles of amplification at 95 °C for 10 sec, 60 °C for 10 sec and 72 °C for 6 sec, followed by a melting curve analysis from 65 °C to 95 °C. A no template control was included on every plate to rule out nonspecific contamination, while the melting curve analysis was included to verify that a single specific product was measured in each run. 

### Data analysis

Reads were aligned to the draft genome of European eel [15] using TopHat (version 2.0.5) [23]. The resulting files were filtered using SAMtools (version 0.1.18) [24] to exclude secondary alignment of reads. Aligned fragments per predicted gene were counted from SAM alignment files using the Python package HTSeq (version 0.5.3p9) [25]. We only considered gene predictions which have been provisionally functionally annotated by Blast2GO (i.e. known eel genes or gene predictions with homologs in other species). In order to make comparisons across samples possible, these fragment counts need to be corrected for the total amount of sequencing performed for each sample. As a correction scaling factor, we employed library size estimates determined using the R/Bioconductor (release 2.11) package DESeq [26]. Read counts were normalized by dividing the raw counts obtained from HTSeq by its scale factor and by transcript length in kilobases. Detailed read coverage for individual genes was extracted from the TopHat alignments using SAMtools. New alignments were generated for re-annotated genes, which were then quantified and normalized as before, using the scaling factors determined for the initial alignments. For each Gene Ontology category, total expression was calculated by summing the normalized expression of all genes annotated with that GO term (based on Blast2GO annotations, [27]).

## Results

### Gene expression profiling

**Figure 1:**
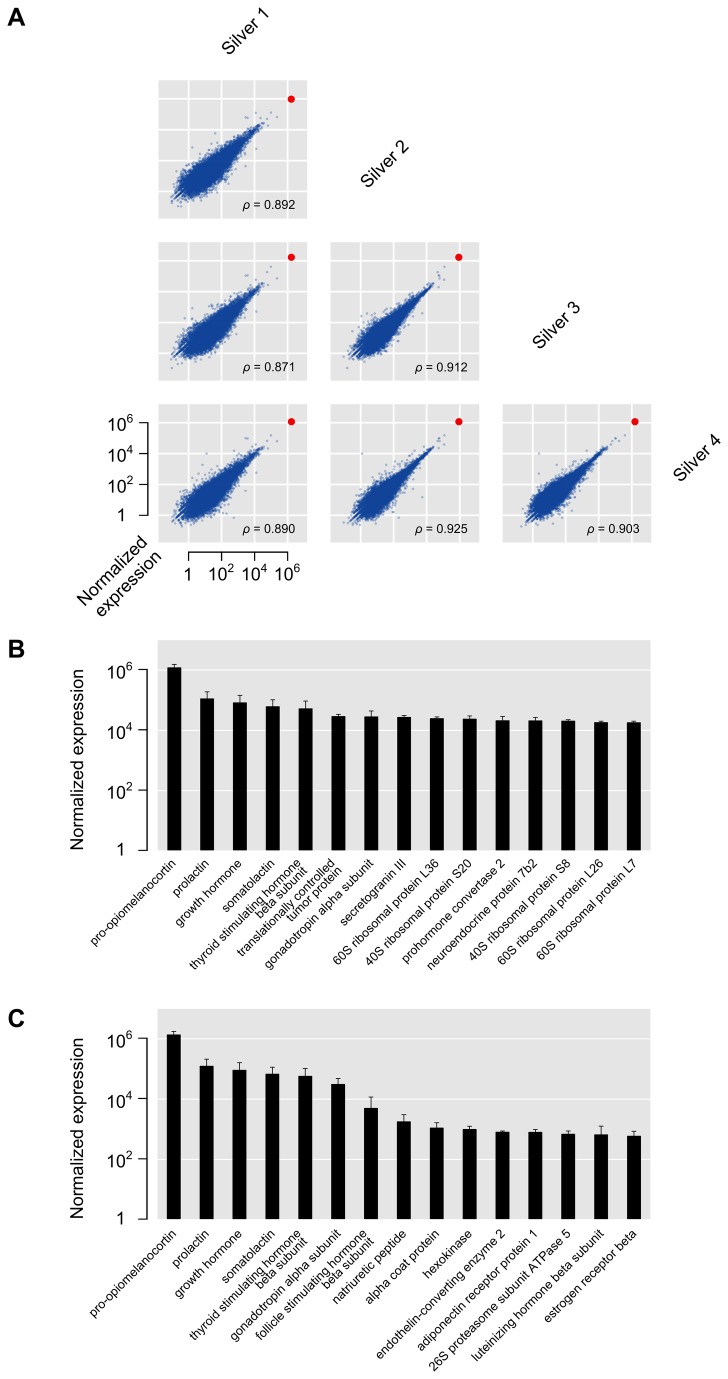
Gene expression in silver eels. (**A**): Pairwise comparisons of normalized expression values for all genes between the four silver eel samples (for details, see Materials and methods). The red dot indicates the expression level of pro-opiomelanocortin. Spearman rank correlations (ρ) for the different comparisons show a good correspondence between the expression values for the different silver eel samples. (**B**): Top 15 expressed genes in silver eel samples, displayed as means of normalized expression ± standard deviations (SD) on a log scale. (**C**): Top 15 expressed genes in GO category Molecular Function ‘hormone activity’ (GO:0005184), displayed as means of normalized expression ± SD on a log scale.

RNA-seq reads were mapped to the European eel genome [15] using TopHat [23]. From the total number of reads that was obtained from the silver eel samples, 90–97% successfully aligned, yielding expression values for 33649 genes with provisional functional annotations. Details about the number of reads and mapping for all samples are given in table S2. Gene expression values for the different silver eel samples are plotted against each other in Figure 1A, and correlate well across all four samples (Spearman rank correlation 0.87–0.93). One particular gene, *pomc*, encoding pro-opiomelanocortin, the precursor for the peptide hormones of the melanocortin system, stands out from the overall expression in all samples as it is expressed at least one order of magnitude higher than any other gene. Further highly expressed genes predominantly encode other hormones and ribosomal proteins (Figure 1B). Figure 1C shows the top genes by expression annotated with the Gene Ontology category ‘hormone activity’ (GO:0005184). All silver eel gene expression values are available as table S3. Several of the genes found to be highly expressed were manually annotated and their sequences submitted to GenBank (see table S4 for details).

### Massive expression of *pomc*

**Figure 2:**
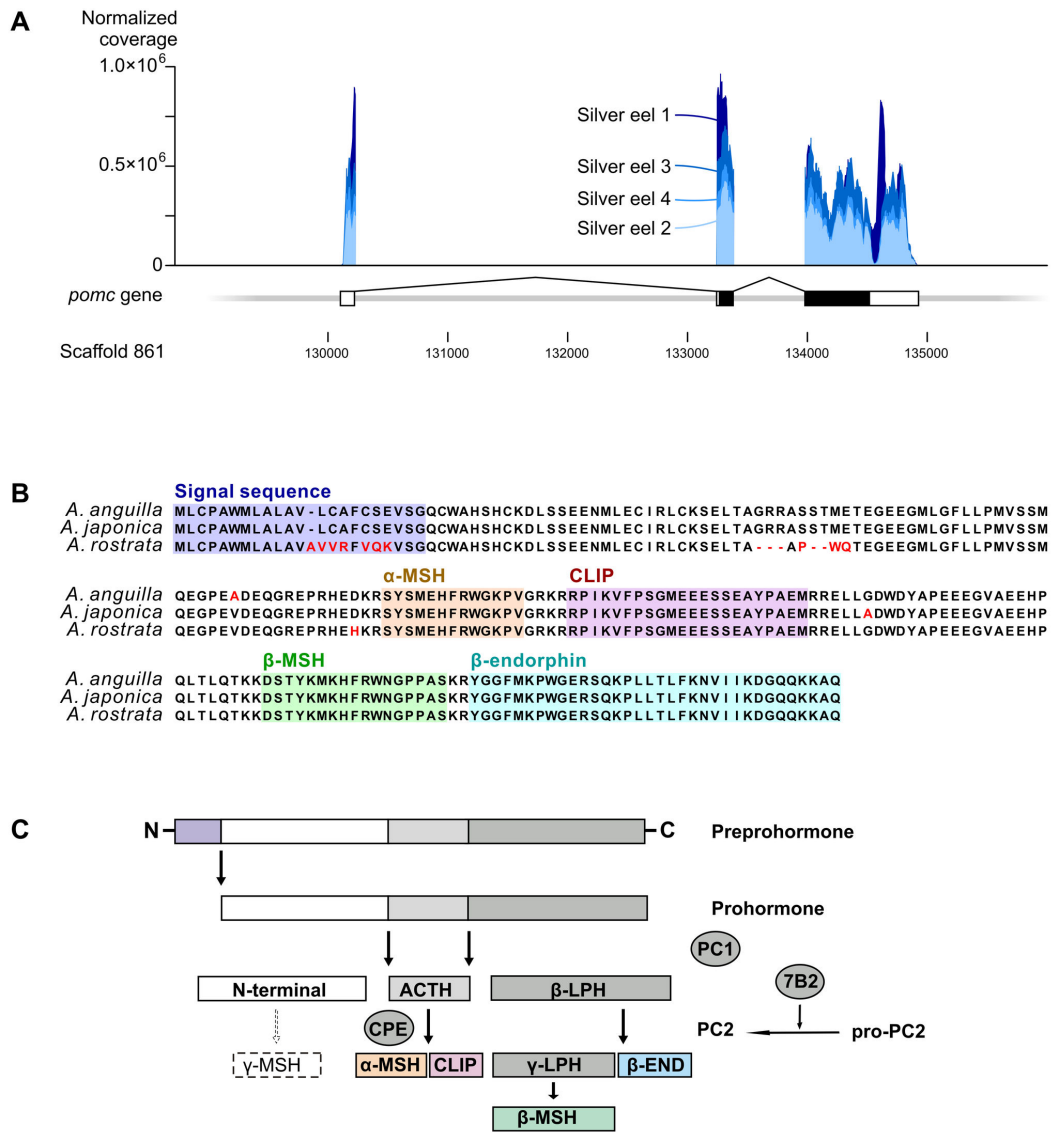
Massive expression of pro-opiomelanocortin in silver eels. (**A**): The gene encoding pro-opiomelanocortin (pomc) was manually annotated based on coverage by RNA-seq reads. *pomc* is located on scaffold 861 of the European eel genome assembly, and contains three exons. The first exon contains the 5′ UTR (white), the second exon contains the last part of the 5′ UTR and the coding sequence (black), while the third exon contains coding sequence and the 3′ UTR (white). The plot indicates the total local read coverage scaled by the normalization factor for each sample and each nucleotide position along the gene. Details about the annotation are given in table S4. A substantial fraction (15–30%) of the total number of aligned reads maps to the *pomc* gene (see table S2 for details). (**B**): Amino acid sequence comparison of *pomc* between different *Anguilla* species. The comparison shows greater similarity between *A. anguilla* (JX441983) and *A. japonica* (AY158010) (99.1%) than between *A. anguilla* and *A. rostrata* (AF194969) (92.3%). (**C**): Post-translational processing of the prohormone Pomc to its bioactive hormone components. In the corticotropes situated in the *rostral* *pars* distalis prohormone convertase 1 (PC1) is known to cleave the prohormone to generate adrenocorticotropic hormone (ACTH) and β-lipotropic hormone (β-LPH), while in the melanotropes in the *pars* *intermedia* these hormones are subsequently cleaved by prohormone convertase 2 (PC2) to generate, respectively, α-melanocyte stimulating hormone (α-MSH) and corticotropin intermediate peptide (CLIP), and β-endorphin (β-END) and γ-lipotropin (γ-LPH, further processed into β-MSH) [42,43,65]. The γ-MSH sequence that derives from the N-terminal part of *pomc* in higher vertebrates is absent from the *pomc* genes of teleosts [66]. The granin neuroendocrine protein 7b2 (7B2) is an endogenous inhibitor protein that is required for active PC2 enzyme. The generation of mature α-MSH is catalysed by carboxypeptidase e (CPE). Figure adapted from [42,67]. The same markings are used for the *pomc* derived peptides in both panel B and C: signal sequence (purple), α-MSH (yellow), CLIP (pink), β-MSH (green) and β-END (blue).

Due to the striking expression of *pomc* we manually improved the annotation of this gene using the RNA-seq alignments and the gene prediction (Figure 2A). Based on the new annotation we also recalculated the expression values, and these are plotted on top of the new annotation in Figure 2A. In the silver eels *pomc* exhibits massive gene expression levels, such that it constitutes up to 30% of the total number of aligned RNA-seq reads mapping to the genome (table S2). Comparison between the gene expression values for the average of the four silver eel samples and yellow eel and mature eel are shown in Figure S1, displaying that the *pomc* gene also exhibits high expression in the two other stages. The amino acid sequences of *pomc* between different *Anguilla* species were compared (Figure 2B), where *A. anguilla* and *A. japonica* display a greater similarity than *A. anguilla* and *A. rostrata*. This is unexpected, given the evolutionary relationships between *Anguilla* species [28]. Figure 2C illustrates the post-translational processing of the prohormone Pomc to its bioactive hormone components, including adrenocorticotropic hormone (ACTH), β-lipotropic hormone (β-LPH), α-melanocyte stimulating hormone (α-MSH), corticotropin intermediate peptide (CLIP), β-endorphin (β-END) and β-melanocyte stimulating hormone (β-MSH). The amino acid sequences of these six bioactive hormones are 100% identical in all three *Anguilla* species (Figure 2B).

### Gene Ontology categorization

**Figure 3:**
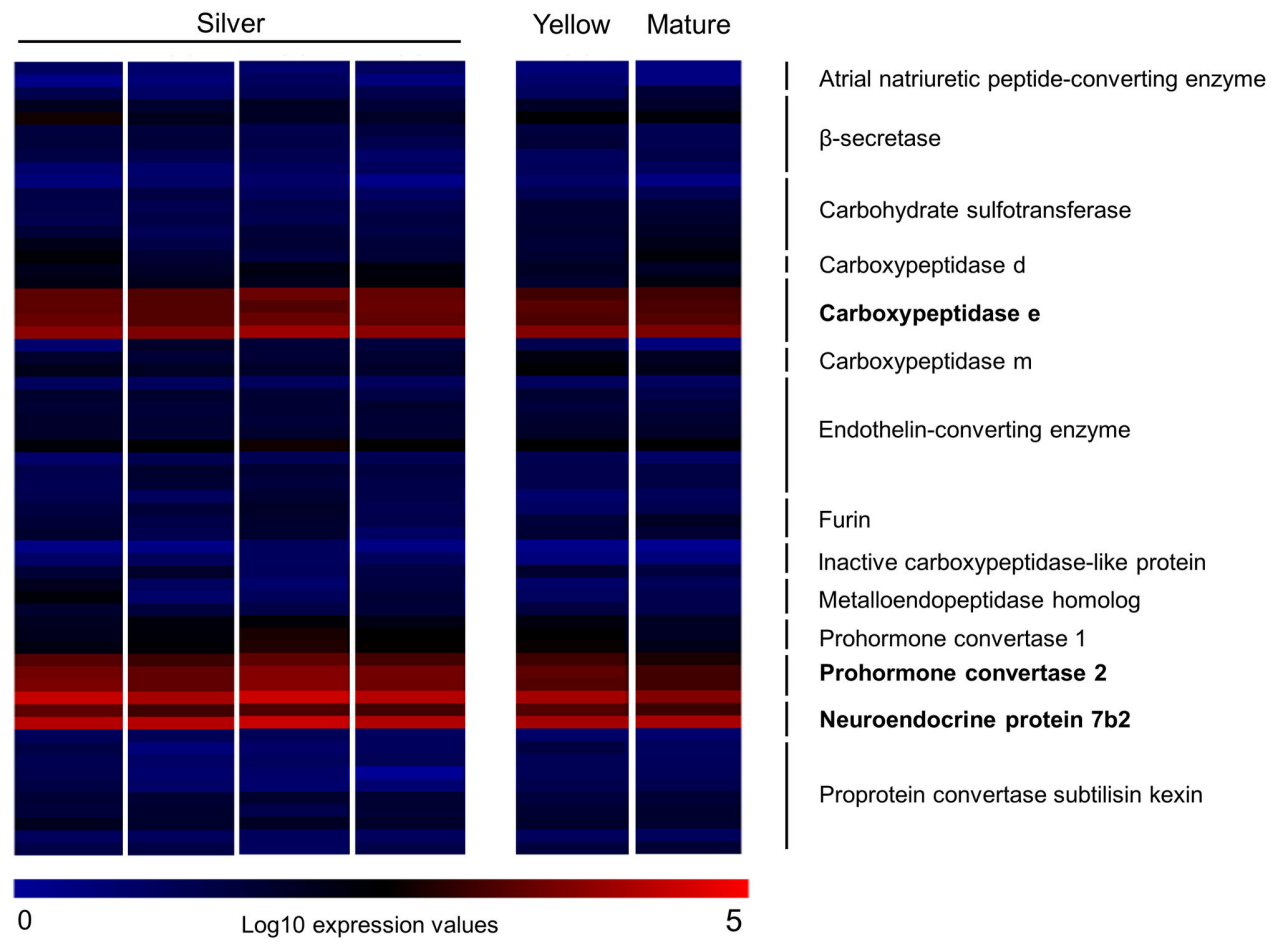
Expression of genes in Gene Ontology category ‘peptide hormone processing’. Expression of genes belonging to the Gene Ontology term ‘peptide hormone processing’ (GO:0016486). The four silver eel samples are grouped together to the left (silver eel 1–4 from left to right), while the gene expression for the yellow eel and the mature eel is placed to the right and displays an expression pattern similar to the silver eel samples. Genes involved in the processing of pro-opiomelanocortin are highly expressed (red), and include carboxypeptidase e, prohormone convertase 2 and neuroendocrine protein 7b2.

In order to investigate the possible involvement of other genes in downstream processing of Pomc, we attempted to look for overrepresentation of certain classes of genes using gene ontology (GO) categories. Gene expression values were summarized for each GO category (based on Blast2GO annotations [27]) of the provisional annotation of the European eel genome [15] (Figure S2). *pomc* is included in all of the top 15 highly expressed categories, stressing the dominance of this gene in the transcriptome. Genes that belong to the GO-category ‘peptide hormone processing’ (GO:00016486) were investigated to look for potential important players of downstream processing of prohormones (like Pomc) into bioactive peptide hormones. This analysis revealed high expression of different players involved in the post-translational processing of Pomc [29–32], including carboxypeptidase e, prohormone convertase 2 and neuroendocrine protein 7b2 (Figure 3). Note that the different convertases and catalysts involved in the processing of the prohormone are included in Figure 2C.

### High expression of Pomc-processing genes

**Figure 4:**
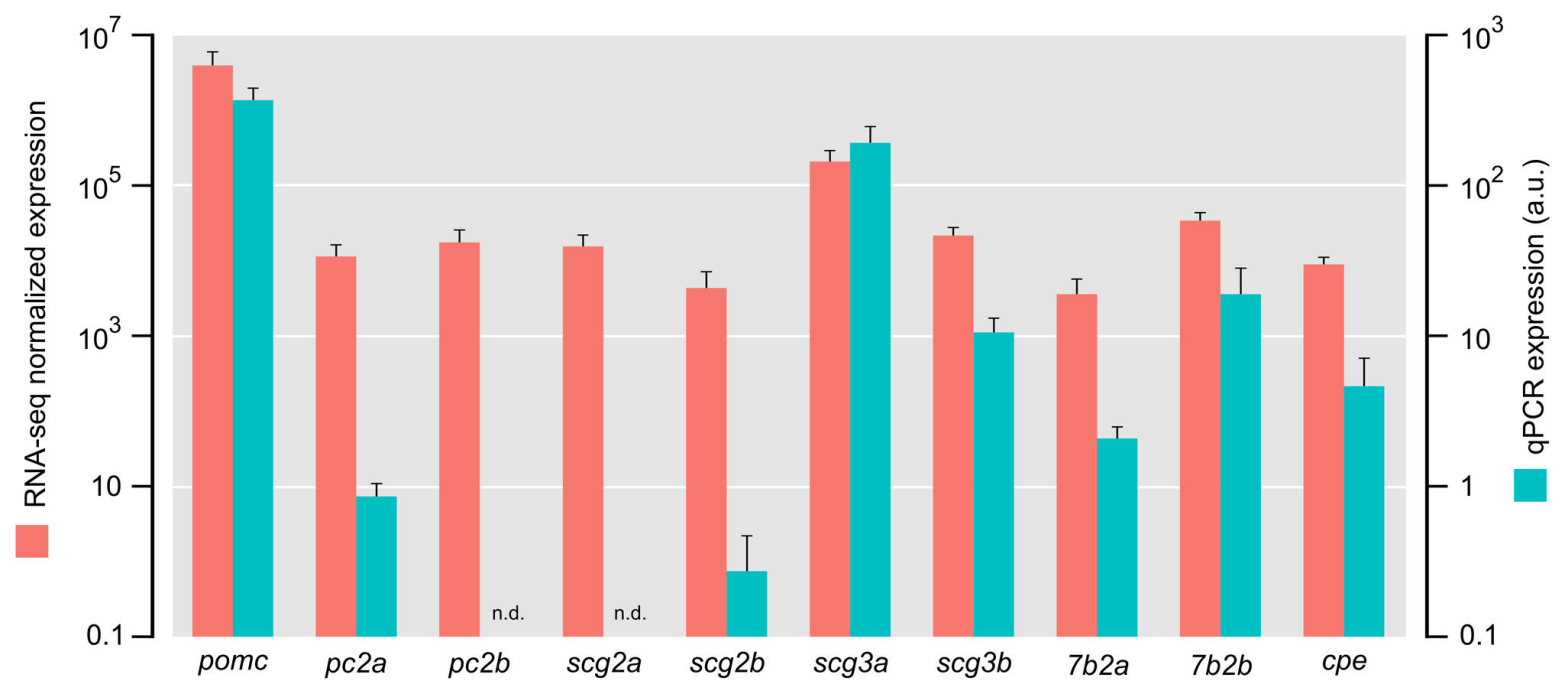
Comparison between RNA-seq and qPCR experiments of genes involved in the melanocortin system. Quantitative PCR validation of pro-opiomelanocortin and other highly expressed genes involved in the processing of the prohormone found by RNA-seq in the silver eel samples: pro-opiomelanocortin (pomc), prohormone convertase 2 copy 1 (pc2a), prohormone convertase 2 copy 2 (pc2b), secretogranin II copy 1 (scg2a), secretogranin II copy 2 (scg2b), secretogranin III copy 1(scg3a), secretogranin III copy 2 (scg3b), neuroendocrine protein 7b2 copy 1(7b2a), neuroendocrine protein 7b2 copy 2 (7b2b) and carboxypeptidase e (cpe). Note that *pc2b* and *scg2a* were studied by RNA-seq only, and not investigated by qPCR due to limitations for designing primers from these genes where we only obtained partial sequences. Primer sequences are given in table S1 and details about the annotation of the genes are provided in table S4. Results are presented as means ± standard deviations (SD) on log scales for both methods, where the left y-axis represent the re-quantified RNA-seq normalized gene expression (n=4) and the right y-axis represent the expression by qPCR (n=5).

Due to the high expression of the gene encoding neuroendocrine protein 7b2 (also called secretogranin V), we decided to investigate if other secretogranins also exhibited high expression. Among the genes of the granin family proteins, known to be associated with neuroendocrine secretion, secretogranin II and secretogranin III coding genes were found to be highly expressed. In particular, one secretogranin III paralogue exhibited substantial gene expression levels (approximately 3% of total reads, see table S2 for details). Based on alignments and gene predictions, the highly expressed genes known to be involved in Pomc processing were manually annotated and re-quantified (Figure 4 and table S4). In nearly all cases, the improved annotation resulted in higher gene expression values (Figure S3). This increase is an effect of both updated gene structure leading to more reads aligning, and of better UTR definition leading to shorter transcript annotations (see the definition of normalized expression in Materials and methods). The high expression of *pomc* and the genes involved in the processing of the prohormone that were found to exhibit high expression by RNA-seq were validated by qPCR (Figure 4), showing a good correlation in relative expression levels between the two different methods.

## Discussion

This study serves as a first exploration of the complete pituitary transcriptome of prepubertal female European silver eel. Using the draft genome and the RNA-seq read coverage we have improved the annotation of the most highly expressed genes expressed in the eel pituitary.

*pomc* was found to be by far the most abundant transcript in the European eel pituitary (figures 1 and 4), eclipsing the expression of all other genes in the pituitary gland (figures 1B, C and figure S2). In teleosts, the pituitary gland consists of multiple hormone-producing cell types, including lactotropes, corticotropes, somatotropes, thyrotropes, two types of gonadotropes, melanotropes and somatolactotropes [33]; however, the *pomc* gene is expected to be expressed only in corticotropes and melanotropes. Since the RNA-seq analysis was performed on complete pituitary glands, in some eels the amount of *pomc* mRNA in these two cell types may very well exceed 50% of total mRNA content. Alternatively, *pomc* could be expressed in other cell types as well. It is worth noting that the very high expression level of *pomc* observed may also be a consequence of the technology employed. Using RNA-seq, the *pomc* transcript was determined to be almost five hundred-fold more abundant than the transcripts of several housekeeping genes (e.g. *b-actin*). Until recently, full transcriptome profiling was performed using microarrays, which generally detect a much more limited range in expression levels (approximately a 1000-fold change between ‘low’ and ‘high’ expression values). If we had used microarrays, we would have been unable to detect the difference in expression level between *pomc* and a housekeeping gene like *b-actin*: both transcripts would probably have saturated the available probes, and would have received the maximum ‘high’ expression value. Strikingly, the top 15 of the GO categories by gene expression all include *pomc*, again emphasizing the total dominance of this prohormone in the silver eel pituitary transcriptome with regards to gene expression.

The eels employed in this study were obtained from several different sources (table 1), resulting in a range of external conditions (salinity, photoperiod, season, animal handling etc.). Nevertheless, all samples show a consistent gene expression profile (Figure 1 and Figure S1). When examined in detail, the expression profile for the *pomc* gene is similar in all four silver eel samples (Figure 2A). The only silver eel (sample 1) that was not obtained from a farm exhibits the highest expression of *pomc*, with a slightly different coverage profile near the 5′ UTR (Figure 2A). We observed the same profile in the (non-replicated) yellow and mature eel samples (sequenced at the same time as silver eel 1), suggesting that the biased coverage profile is an artifact of different sequencing technology generations rather than a biological difference. Because of limited availability of high quality samples from non-silver eels, we were unable to study ontogenetic changes in gene expression completely throughout maturation. In addition, the very high expression of just a single gene can make exact comparisons of RNA-seq results for all other genes less robust [34]. However, in the four silver eel replicates, the levels of *pomc* itself correlate positively with body weight and gonadal development (GSI) (table 1).

The animals could have been stressed by handling, transport, or experimental procedures prior to the pituitary dissection, which conceivably could affect downstream gene expression results, particularly of *pomc*. ACTH (corticotropin) produced downstream of pro-opiomelanocortin is an important player in the neuroendocrine stress response which activates cells in the interrenal tissue to produce and release cortisol [35]. However, the abundance of *pomc* was high for all the samples regardless of the different sources they came from. Farmed animals are more used to handling than wild animals, which possibly could influence how well they cope with stress. The transport of these animals (see Materials and methods) may have induced additional stress, although the kinetics of mRNA synthesis imply that by itself this interval is too brief to result in the levels of *pomc* expression observed [36]. The silver eel that was caught in the wild exhibited the highest *pomc* expression compared to the farmed silver eels, while the artificially matured eel that received injections every seven days for 17 weeks was the sample that exhibited the lowest *pomc* expression levels of all the samples. These observations suggest that it is unlikely that the high abundance of *pomc* can be explained by induction of stress during animal handling only. Nevertheless, a previous stressed state (either induced by handling or by biological factors) cannot be excluded, and the possibility should be kept in mind when interpreting the results. It would be difficult to obtain demonstrably non-stressed eels, which would be needed to create a baseline for a stress marker (i.e. cortisol) that would make it possible to compare with the levels of other animals. In fact, in the few studies in which cortisol levels were determined in eels, these were found to be strongly elevated, yet highly variable, in migrating silver eels [37,38]. 

The activity of the Pomc-producing cells is regulated by expression and cleavage of the precursor protein, post-translational processing of cleavage products, and release of the end products (Figure 2C). In mammals post-translational processing of Pomc is dependent on the proteolytic cleavage by prohormone convertases (PC1 and PC2; for review see 39), which are most likely also involved in the processing of fish Pomc [40]. PC1 mediates the initial processing of Pomc into ACTH, β-LPH and N-terminal peptide in the corticotropes of the pituitary *rostral pars distalis*, while in the melanotropes located in the pituitary *pars intermedia*, PC2 processes ACTH further into α-MSH and corticotropin-like intermediate peptide (CLIP), and converts β-LPH into β-MSH (processed via γ-LPH) and β-endorphin [30,41,42]. Carboxypeptidase e (CPE) catalyzes the generation of mature α-MSH from ACTH by trimming the C-terminal, and also works as a sorting receptor of the regulated secretory pathway by binding secretory proteins, including Pomc [29,43]. In our data, *pc1*, *pc2* and *cpe* were among the highly expressed genes, with *pc2* showing higher expression than *pc1* (Figure 3). The high expression of these genes implies an important role for the processing of Pomc and emphasizes that the dramatically high expression of *pomc* is likely to be biologically relevant (Figure 3).

PC2 has a specific endogenous inhibitor called granin neuroendocrine protein 7b2 (7B2), which functions as a chaperone protein and is required for production of active PC2 enzyme [31,44]. The catalytic activity of 7B2 is regulated by inhibiting PC2 unfolding and aggregation in the secretory vesicle [32]. 7B2 is a member of the granin family, which includes biologically active peptides that are responsible for delivery of peptides, hormones, neurotransmitters and growth factors. These proteins are expressed in endocrine cells and peptidergic neurons and have both constitutive and regulated secretory pathways [45]. Secretogranin II (Scg2) can be proteolytically processed to generate secretoneurin [46,47]. In goldfish gonadotropes secretoneurin has been shown to stimulate luteinizing hormone synthesis and release [48]. Scg3 can be cleaved to peptides in secretory vesicles [49]. Scg3 and CPE have been found to interact and facilitate prohormone sorting within secretory granules [50]. Pomc and Scg3 have been found to coordinately increase upon stimulation of *Xenopus* pituitary *pars intermedia* cells *in vivo* [51]. In the silver eel pituitary, several members of the granin family show high expression levels (figures 3 and 4). In contrast to other studied teleosts, the European eel has likely retained two *scg3* paralogues after the teleost specific genome duplication. These differ markedly in expression in the eel pituitary, indicative of possible subfunction partitioning [52]. One *scg3* paralogue exhibits very high expression in the silver eel pituitary (Figure 4), suggesting an important role for this granin in the secretory pathway in eel.

The biologically active peptide hormones derived from *pomc* exert a variety of physiological functions in fish, including effects on stress, vasoregulation, thermoregulation, growth, metabolism, metamorphosis and reproduction (for review see 53). Several studies have indicated involvement of the melanocortin system in the regulation of energy metabolism and food intake in fish [53–56]. It has been suggested to control the energy balance by decreasing food intake and enhancing energy costs. α-MSH has been shown to stimulate lipase activity and increase the circulating levels of fatty acids in rainbow trout, while trout with defective α-MSH show increased appetite, enlarged livers and accumulation of fat in the abdominal cavity [57]. Synchronous changes in gonadal development and morphological characteristics (e.g. skin coloration), have been demonstrated in European and Japanese eel and are suggested to be hormonally regulated [20,58]. Degeneration of the gut takes place during gonadal maturation in European eel [59]. Eels have an exceptionally high fat content prior to migration [60], which suggests a role for Pomc-derived peptides in fat metabolism. Adaptation to background color is an important function found to be regulated by α-MSH (reviewed in 54), where regulation of skin pigmentation mediated by α-MSH exerts actions opposing those of melanin-concentrating hormone (MCH) [55,61]. The high *pomc* expression levels might reflect an important role for α-MSH in the changes in skin coloration occurring during silvering in eel.

In light of these physiological adaptations mediated by the melanocortin system, an alternative explanation of potential stress (see discussion above) can develop, in which silver eels naturally exhibit the characteristics of a stressed state and therefore experience a chronic activation of the Pomc-dependent stress response. For example, the high levels of cortisol observed in silver eels have been interpreted as being involved in the mobilization of energy (fat stores) and the adaptation to seawater [62]. In addition, cortisol stimulates the expression of the luteinizing hormone β-subunit in European eel [63], thus providing a more complex picture of cortisol regulation, in which cortisol does not only negatively affect reproduction due to stress, but can also be beneficial for the induction of sexual maturation. 

From this initial survey of the eel pituitary transcriptome it is not possible to precisely disentangle the relative contributions of prior biological stress-like processes and stress induced by the experiment. This would require much more comprehensive sampling, including additional biological states, points in time, and well-defined stressors. Considering the dominance of comparatively few genes in the reported trancriptomes, the lack of detailed physiological clues in the single *pomc* transcript species, and the relatively high cost of full transcriptome sequencing, targeted proteomics techniques would be well suited for such an experiment. A recent study utilized mass spectrometry analyses to reveal the post-translational processing of *pomc* in the pituitary of medaka (*Oryzias latipes*) [64]. The availability of the draft genome of European eel and the detailed annotation of the central genes we supply in the current paper makes it possible to predict the molecular weights of all protein products, which facilitates the use of mass spectrometry. 

Although more investigation is required to reveal the mechanisms by which the melanocortin system is involved in processes such as growth and metabolism, reproduction, water balance, and body pigmentation in teleosts, the results presented here support the idea that the control of this system is a major function of the eel pituitary. 

## Supporting Information

Figure S1 **Gene expression in all samples.** Differences in normalized gene expression between the average expression values for the four silver eel samples compared to the yellow eel and mature eel samples. The red dot indicates the expression level of pro-opiomelanocortin. Spearman rank correlations (ρ) for the different comparisons show a good correspondence between the expression values for the different samples.
(TIF)

Figure S2 **Gene Ontology characterization.** For each GO category the total expression for the average of the silver eel samples was calculated by summing the normalized expression of all genes annotated with that GO category. The 25 most highly expressed GO categories based on expression values in the silver eel samples are displayed (for details, see Materials and methods), of which the top 15 categories include *pomc*, underlining the dominance of this gene. The red line corresponds to *pomc* gene expression alone (based on the original annotation). The first category by expression that does not include *pomc* is ‘protein binding’.
(TIF)

Figure S3 **The importance of re-annotation of genes for gene expression in silver eel samples.** Differences in gene expression values for the four silver eel samples, highlighting the genes involved in the melanocortin system that were re-annotated in this study. The original gene expression values (before re-annotation) are shown in red and the new gene expression values (after re-annotation) are displayed in green. The figure illustrates the high expression of the genes involved in the melanocortin system as compared to the overall gene expression (grey), and that re-calculation of gene expression values after re-annotation of these genes increases their relative gene expression in all silver eel samples.
(TIF)

Table S1 **Primer sequences used for qPCR in this study.**
(DOCX)

Table S2 **Alignment of RNA-Seq reads.**
(DOCX)

Table S3 **Expression values.**
(XLSX)

Table S4 **Annotation of highly expressed genes.**
(DOCX)
