# Supplementary figures and images for: The Pituitary Gland of the European Eel Reveals Massive Expression of Genes Involved in the Melanocortin System
Source: PLoS One. 2013 Oct 10;8(10):e77396. doi: 10.1371/journal.pone.0077396 (PMC3795071; doi:10.1371/journal.pone.0077396)

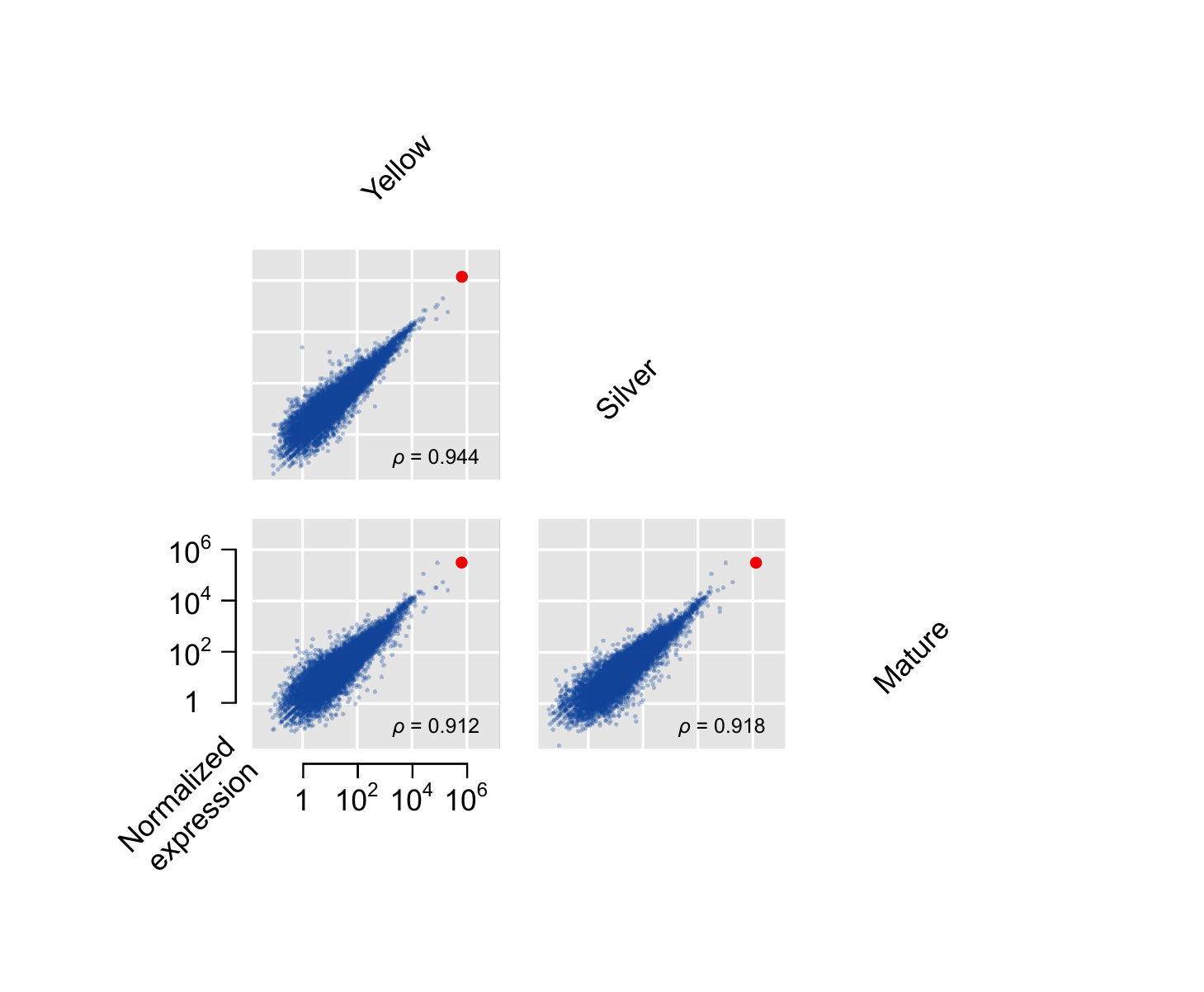

Supplement: Figure S1 — Gene expression in all samples. Differences in normalized gene expression between the average expression values for the four silver eel samples compared to the yellow eel and mature eel samples. The red dot indicates the expression level of pro-opiomelanocortin. Spearman rank correlations (ρ) for the different comparisons show a good correspondence between the expression values for the different samples. (TIF) [file pone.0077396.s001.tif]

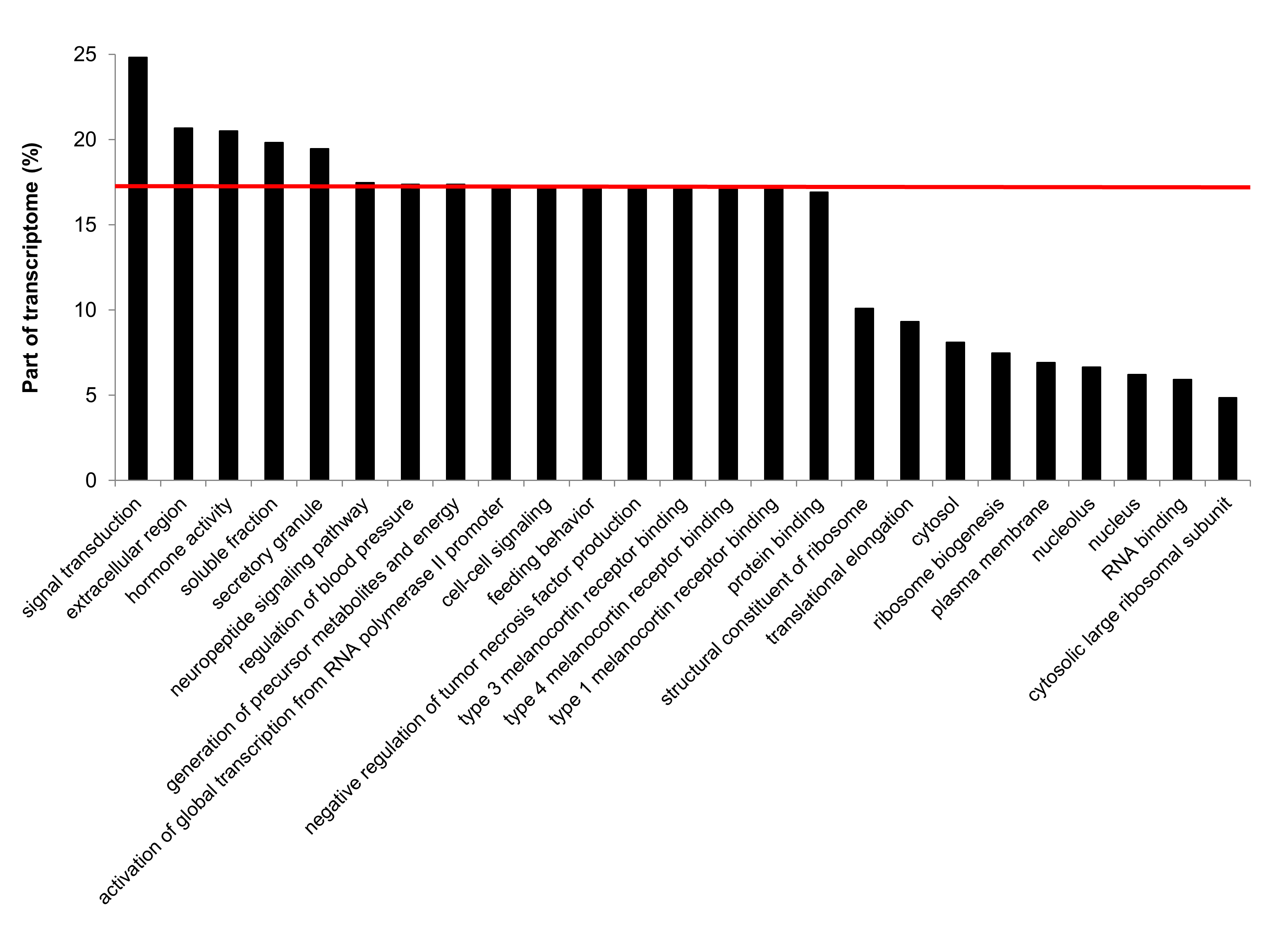

Supplement: Figure S2 — Gene Ontology characterization. For each GO category the total expression for the average of the silver eel samples was calculated by summing the normalized expression of all genes annotated with that GO category. The 25 most highly expressed GO categories based on expression values in the silver eel samples are displayed (for details, see Materials and methods), of which the top 15 categories include pomc, underlining the dominance of this gene. The red line corresponds to pomc gene expression alone (based on the original annotation). The first category by expression that does not include pomc is ‘protein binding’. (TIF) [file pone.0077396.s002.tif]

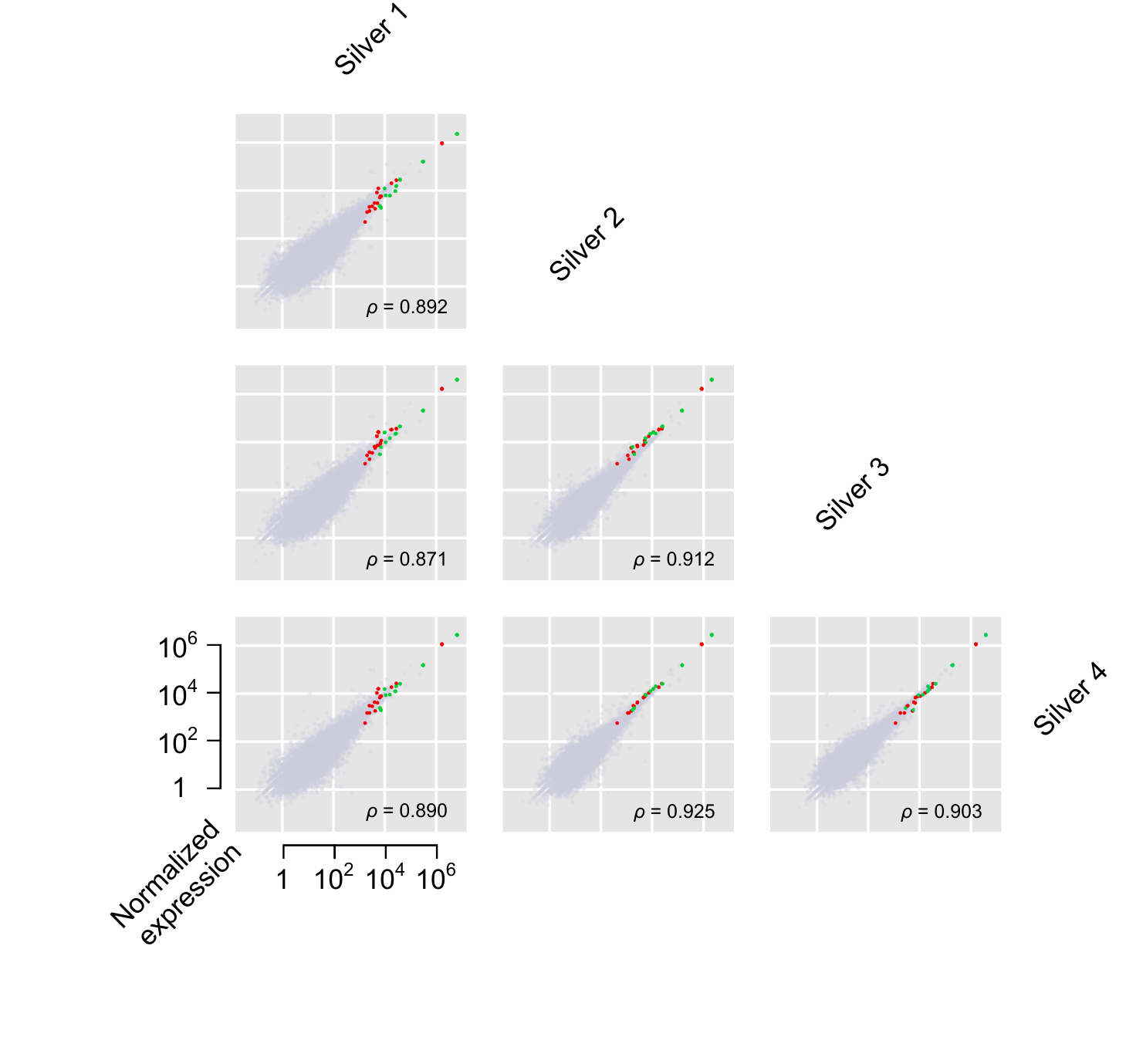

Supplement: Figure S3 — The importance of re-annotation of genes for gene expression in silver eel samples. Differences in gene expression values for the four silver eel samples, highlighting the genes involved in the melanocortin system that were re-annotated in this study. The original gene expression values (before re-annotation) are shown in red and the new gene expression values (after re-annotation) are displayed in green. The figure illustrates the high expression of the genes involved in the melanocortin system as compared to the overall gene expression (grey), and that re-calculation of gene expression values after re-annotation of these genes increases their relative gene expression in all silver eel samples. (TIF) [file pone.0077396.s003.tif]
