# Supplementary material for: The Pituitary Gland of the European Eel Reveals Massive Expression of Genes Involved in the Melanocortin System
Source: PLoS One. 2013 Oct 10;8(10):e77396. doi: 10.1371/journal.pone.0077396 (PMC3795071; doi:10.1371/journal.pone.0077396)
Supplement: Table S1 — Primer sequences used for qPCR in this study. (DOCX) [file pone.0077396.s004.docx]

**Table S1**. Primer sequences used for qPCR in this study.

| Target | Primer sequence | Amplicon size (nt) | Efficiency |
| --- | --- | --- | --- |
| *pomc* | F 5’-TTCAGAGGTGAGCGGTCAGT-3’  R 5’-TTCAGACTTGCACAGGCGTA-3’ | 97 | 2.04 |
| *pc2a* | F 5’-GCTACACGGACGACTGGTTT-3’  R 5’-CACCGCAGATGTTGTTGTTC-3’ | 84 | 1.96 |
| *scg2b* | F 5’-GAGATGCGATACACCCACAA-3’  R 5’-GATGGGACTGCATCCTCTGT-3’ | 63 | 2.07 |
| *scg3a* | F 5’-GGAAAGACCACTGCAAGAGC-3’  R 5’-CCCGGTCTATTTCTGCCTCT -3’ | 107 | 2.00 |
| *scg3b* | F 5’-AAAGATGGCAAGCAAGAGGA-3’  R 5’-CTTGATGACCTCACCCTCGT-3’ | 117 | 2.05 |
| *7b2a* | F 5’-GCTGGCTAAGTGGAACAAGG-3’  R 5’-CAATCTCTGGCCCATCAAAT-3’ | 100 | 2.00 |
| *7b2b* | F 5’-GAACGGTGTGCAGGAGAAA-3’  R 5’-GTGTGTACCACCCTGGCTGT-3’ | 95 | 1.99 |
| *cpe* | F 5’-CCGGGAACTACAAAGTGTCG-3’  R 5’-CTCCACCAACGACTCCAAGT-3’ | 116 | 1.97 |
| *arp* | F 5’-GTGCCAGCTCAGAACACTG-3’  R 5’-ACATCGCTCAAGACTTCAATGG-3’ | 107 | 1.97 |
