# Supplementary material for: The Pituitary Gland of the European Eel Reveals Massive Expression of Genes Involved in the Melanocortin System
Source: PLoS One. 2013 Oct 10;8(10):e77396. doi: 10.1371/journal.pone.0077396 (PMC3795071; doi:10.1371/journal.pone.0077396)
Supplement: Table S2 — Alignment of RNA-Seq reads. (DOCX) [file pone.0077396.s005.docx]

**Table S2.** Alignment of RNA-Seq reads.

Aligned reads to the *A. anguilla* genome using TopHat and quantifications of the aligned reads using HTseq. The alignments to the original pro-opiomelanocortin (*pomc*) and secretogranin III copy 1 (*scg3a*) are included, as well as the new alignments after the manual re-annotation of these genes. Percentages of aligned reads are relative to the total sequenced number of reads. All other percentages are relative to the number of aligned reads.

|  | **Silver eel 1** | **Silver eel 2** | **Silver eel 3** | **Silver eel 4** | **Yellow eel** | **Mature eel** |
| --- | --- | --- | --- | --- | --- | --- |
| Reads | 2 x 34 979 211 | 2 x 12 868 247 | 2 x 8 115 788 | 2 x 8 265 310 | 2 x 32 610 312 | 2 x 29 733 953 |
| Aligned reads^1^ | 31 559 019  (90.2%) | 12 437 924  (96.7%) | 7 834 849  (96.5%) | 8 036 605  (97.2%) | 29 400 983  (90.2%) | 24 688 357  (83.0%) |
| Quantified reads, original | 22 147 534  (70.2%) | 8 591 952  (69.1%) | 5 625 555  (71.8%) | 5 629 873  (70.1%) | 19 877 926  (67.6%) | 16 646 953  (67.4%) |
| Quantified reads, annotated only^2^ | 19 797 190  (62.7%) | 7 748 716  (62.3%) | 5 144 679  (65.7%) | 5 153 350  (64.1%) | 17 179 534  (58.4%) | 13 759 075  (55.7%) |
| Original *pomc* alignments | 7 264 700  (23.0%) | 1 753 463  (14.1%) | 1 598 406  (20.4%) | 1 345 307  (16.7%) | 3 271 467  (11.1%) | 1 411 832  (5.7%) |
| New *pomc* alignments | 9 506 165  (30.1%) | 2 174 308  (17.5%) | 1 956 854  (25.0%) | 1 675 734  (20.9%) | 4 334 693  (14.7%) | 1 900 682  (7.7%) |
| Original *scg3a* alignments | 469 540  (1.5%) | 164 911  (1.3%) | 112 601  (1.4%) | 100 525  (1.3%) | 516 365  (1.8%) | 314 118  (1.3%) |
| New *scg3a* alignments | 1 091 385  (3.5%) | 361 987  (2.9%) | 237 805  (3.0%) | 216 715  (2.7%) | 1 069 095  (3.6%) | 644 190  (2.6%) |

^1^ Where at least one read of the pair aligns

^2^ Reads with no functional annotation removed from original alignments
