# Supplementary material for: The Pituitary Gland of the European Eel Reveals Massive Expression of Genes Involved in the Melanocortin System
Source: PLoS One. 2013 Oct 10;8(10):e77396. doi: 10.1371/journal.pone.0077396 (PMC3795071; doi:10.1371/journal.pone.0077396)
Supplement: Table S4 — Annotation of highly expressed genes. (DOCX) [file pone.0077396.s007.docx]

**Table S4**. Annotation of highly expressed genes.

| **Gene** | **Predicted gene*** | **mRNA (bp)** | **Protein (aa)** | **Number of exons** | **Best tblastx hit (NCBI non-redundant)** | **Identity/ similarity (%)**  **(blast)** | **E-value (blast)** | **GenBank accession number** |
| --- | --- | --- | --- | --- | --- | --- | --- | --- |
| Pro-opiomelanocortin  (*pomc*) | g8378 g8379 | 1202 | 220 | 3 | *Anguilla japonica* proopiomelanocortin AY158010.1 | 99%/99% | 0E00 | JX441983 |
| Prohormone convertase 2 copy 1  (*pc2a*) | g4316 g4317 | 4699 | 640 | 12 | *Salmo salar* Neuroendocrine convertase 2 BT045314.1 | 85%/91% | 0E00 | JX441984 |
| Prohormone convertase 2 copy 2  (*pc2b*) | g21571 g22548 g22549 | 1176 (partial) | 334 (partial) | 9 | *Salmo salar* Neuroendocrine convertase 2 BT045314.1 | 91%/96% | 0E00 | JX441985 |
| Secretogranin IIa  (*scg2a*) | g38980 g39957 | 972 (partial) | 324 (partial) | 1 | *Ctenopharyngodon idella* Secretogranin II AY634230 | 67%/86% | 4E-46 | JX441986 |
| Secretogranin IIb  (*scg2b*) | g10799 | 2256 | 596 | 2 | *Salmo salar* Secretogranin-2 BT059470 | 48%/64% | 6E-44 | JX441987 |
| Secretogranin III copy 1  (*scg3a*) | g592 g593 | 2863 | 475 | 12 | *Danio rerio* Secretogranin III NM_200757.1 | 68%/83% | 1E-146 | JX441988 |
| **Gene** | **Predicted gene*** | **mRNA (bp)** | **Protein (aa)** | **Number of exons** | **Best tblastx hit (NCBI non-redundant)** | **Identity/ similarity (%)**  **(blast)** | **E-value (blast)** | **GenBank accession number** |
| Secretogranin III copy 2  (*scg3b*) | g9352 9353 | 2853 | 470 | 12 | *Danio rerio* Secretogranin III NM_200757.1 | 77%/86% | 1E-168 | JX441989 |
| Neuroendocrine protein 7b2 copy 1  (*7b2a*) | g1682 | 1866 | 209 | 6 | *Acipenser sinensis* Neuroendocrine protein (7B2) HQ634259.2 | 79%/90% | 4E-88 | JX441990 |
| Neuroendocrine protein 7b2 copy 2  (*7b2b*) | g6117 | 2441 | 220 | 6 | *Oreochromis niloticus* Neuroendocrine protein 7B2-like, XM_003446267.1 | 93%/96% | 1E-116 | JX441991 |
| Carboxypeptidase e  (*cpe*) | g6941 g31187  g32801 | 2640 (partial) | 367 (partial) | 8 | *Oreochromis niloticus* prohormone-processing carboxypeptidase E XM_003449689 | 90%/97% | 0E00 | JX441992 |

* Original genes from the draft genome of the European eel predicted by Henkel et al. 2012 [15].
